# Naïve Learners Show Cross-Domain Transfer after Distributional Learning: The Case of Lexical and Musical Pitch

**DOI:** 10.3389/fpsyg.2016.01189

**Published:** 2016-08-08

**Authors:** Jia Hoong Ong, Denis Burnham, Catherine J. Stevens, Paola Escudero

**Affiliations:** The MARCS Institute for Brain, Behaviour and Development, Western Sydney University, SydneyNSW, Australia

**Keywords:** cross-domain transfer, distributional learning, statistical learning, language and music, pitch

## Abstract

Experienced listeners of a particular acoustic cue in either speech or music appear to have an advantage when perceiving a similar cue in the other domain (i.e., they exhibit cross-domain transfer). One explanation for cross-domain transfer relates to the acquisition of the foundations of speech and music: if acquiring pitch-based elements in speech or music results in heightened attention to pitch in general, then cross-domain transfer of pitch may be observed, which may explain the cross-domain phenomenon seen among listeners of a tone language and listeners with musical training. Here, we investigate this possibility in naïve adult learners, who were trained to acquire pitch-based elements using a distributional learning paradigm, to provide a proof-of-concept for the explanation. Learners were exposed to a stimulus distribution spanning either a Thai lexical tone minimal pair or a novel musical chord minimal pair. Within each domain, the distribution highlights pitch to facilitate learning of two different sounds (Bimodal distribution) or the distribution minimizes pitch so that the input is inferred to be from a single sound (Unimodal distribution). Learning was assessed before and after exposure to the distribution using discrimination tasks with both Thai tone and musical chord minimal pairs. We hypothesize: (i) distributional learning for learners in both the tone and the chord distributions, that is, pre-to-post improvement in discrimination after exposure to the Bimodal but not the Unimodal distribution; and (ii) for both the tone and chord conditions, learners in the Bimodal conditions but not those in the Unimodal conditions will show cross-domain transfer, as indexed by improvement in discrimination of test items in the domain *other* than what they were trained on. The results support both hypotheses, suggesting that distributional learning is not only used to acquire the foundations of speech and music, but may also play a role in cross-domain transfer: as a result of learning primitives based on a particular cue, learners show heightened attention to that cue in any auditory signal.

## Introduction

Language and music are two human universals that share many commonalities. For instance, both consist of discrete elements which are organized in a hierarchical structure governed by rules or syntax (Patel, 2003): in language, phonemes are organized into syllables, which are then organized into words and sentences; in music, notes are organized into chords, which then comprise musical phrases. Furthermore, spoken language (speech) and music rely on similar acoustic dimensions in their expression such as rhythm, pitch, intensity, and timbre—although the relative importance of each dimension depends on the language or music system in question (Patel, 2008).

Given the many commonalities between the two, the question arises whether having experience in one domain may be advantageous for perceiving and learning the other domain, that is, whether cross-domain transfer is possible. There is evidence of music-to-speech transfer in various aspects of language (Besson et al., 2011) such as syllabic perception (Musacchia et al., 2007; Ott et al., 2011; Elmer et al., 2012; Kühnis et al., 2013; Chobert et al., 2014; Bidelman and Alain, 2015), phonological ability (Slevc and Miyake, 2006; Herrera et al., 2011) and word segmentation (François et al., 2012). Furthermore, musicians show higher performance relative to non-musicians in identifying and discriminating lexical tones—a linguistic device used in tone languages in which meaning can be differentiated by a constellation of cues, primarily pitch (e.g., in Mandarin, /ma55/^1^ refers to ‘mother’ while /ma51/ refers to ‘to scold’; Yip, 2002). The advantage musicians enjoy is seen both at the level of behavior (Alexander et al., 2005; Wong and Perrachione, 2007; Burnham et al., 2014) and electrophysiology (Wong et al., 2007; Marie et al., 2011). Since musicians generally have extensive training in perceiving, producing, and attending to musical pitch, it appears that they are able to transfer their expertise with musical pitch to perceive linguistic pitch.

Transfer also occurs in the other direction, that is, extensive experience with a particular speech cue can be transferred to other non-speech/music domains. For example, native Finnish listeners, whose language is a quantity language (i.e., it distinguishes words based on vowel duration), are more accurate at detecting durational deviants of non-speech sounds than native French listeners, whose language does not use duration to distinguish meaning (Marie et al., 2012). In terms of musical pitch, tone language listeners are more successful than non-tone language listeners in discriminating and producing sung intervals (Pfordresher and Brown, 2009); in tracking pitch of non-speech stimuli that have similar pitch contours to their native lexical tones in the brainstem (Bidelman et al., 2011); and in melody discrimination and pitch memory tasks (Alexander et al., 2011; Bidelman et al., 2013). The evidence suggests that cross-domain transfer is seen following extensive experience with an acoustic cue shared by both speech and music.

Several explanations have been proposed to account for cross-domain transfer (for a review, see Asaridou and McQueen, 2013). One such possible explanation, or at least, for music-to-speech transfer, relates to the sharpening of shared auditory skills honed by extensive musical training, which may lead to musicians having a ‘better ear’ or generally better encoding of the input (Kraus and Chandrasekaran, 2010; Skoe and Kraus, 2012). Another possibility relates to one of the learning mechanisms thought to be used in the acquisition of language and music, namely, statistical learning (Patel, 2008). Statistical learning refers to a domain-general learning mechanism in which learners acquire knowledge through extracting statistical regularities in the input. Cross-domain transfer may, result from such learning: while acquiring aspects of language or music that uses a particular acoustic cue (e.g., acquiring pitch-based elements such as lexical tones or musical pitch), learners may be more likely to show heightened attention to that cue in general. In relation to pitch, musicians and tone language listeners show an advantage in perceiving lexical tones and musical pitch, respectively, since both groups have experience in statistical learning of pitch-based elements and therefore are more likely to attend to pitch in *any* given input than listeners who may not have pitch experience. While it appears that non-musicians, too, use statistical learning to acquire implicit musical knowledge in their native musical system (e.g., Tillmann and McAdams, 2004; Bigand and Poulin-Charronnat, 2006; Stevens et al., 2013), non-musicians may not show cross-domain transfer relative to musicians presumably because non-musicians do not engage in statistical learning of musical pitch to the same extent as musicians. Evidence suggests that the active, purposeful nature of musical training in perceiving and producing musical pitch that musicians experience has consequences for acquiring musical knowledge more efficiently as opposed to listeners who acquire musical knowledge passively (Trainor et al., 2012).

One way to test the possibility that acquisition of speech and music plays a role in cross-domain transfer is by examining whether *naïve* participants show cross-domain transfer of pitch after acquiring either lexical tones or musical pitch via distributional learning, mirroring the phenomenon exhibited by tone language listeners and musicians. Distributional learning is a specific form of statistical learning that refers to acquisition of knowledge based on the distributional structure of the input encountered by the learner. Consider the case of first language acquisition: infants attend to speech sounds that they encounter in their linguistic environment and form phonological categories based on those speech sounds. For simplicity, focusing just on a continuum of voice-onset time (VOT, the duration between the release of the stop and the vibration of the vocal folds) along the range of -120 ms to +20 ms, Hindi-learning infants will likely encounter speech sounds that can be modeled as two normal distributions with one peak at around -85 ms and another at around 13 ms (Lisker and Abramson, 1964). English-learning infants, on the other hand, will encounter speech sounds along the same continuum that can be modeled as a single normal distribution with a peak at around 0 ms. This difference in speech sound distributions—in particular, where the peaks occur—may explain the formation of two voicing categories by Hindi listeners (pre-voiced and voiceless) and the formation of only one voicing category by English listeners (voiceless) along that range of VOT. Distributional learning is used to account for the acquisition of the building blocks of speech and music (e.g., Maye and Gerken, 2000; Maye et al., 2002, 2008; Wanrooij et al., 2013; Escudero and Williams, 2014; Ong et al., 2015, 2016). While it remains to be seen whether distributional learning is generalizable to signals from a different domain, there are reason to believe that this is possible: naïve learners are able to generalize distributional learning to the same acoustic cue in a different context (Maye et al., 2008) and to different speakers (Escudero et al., 2011; Escudero and Williams, 2014) albeit within the same domain (i.e., speech). Evidence also suggests that listeners can apply a phonological principle based on one class of phonemes in their native language (e.g., short vs. long vowels) to perceive another class of phonemes (e.g., short vs. long consonants), even when the listener’s native language does not use consonantal duration phonemically (Pajak and Levy, 2014). If acquiring pitch-based elements (lexical tones or musical pitch) heightens learners’ attention to pitch in general, then we may expect learners to generalize such knowledge to a signal of a different domain, even if it is a different class of pitch distinction—such as a pitch contour distinction between lexical tones (mid-level tone vs. rise–fall tone) and a pitch height difference between musical pitch.

In this study, we used a distributional learning paradigm that consisted of naïve adult learners’ exposure to a distribution that spans either a lexical tone or a musical chord minimal pair. Within each domain, the distributions used could either facilitate the learning of two distinct sounds based on an acoustic cue that differentiates that minimal pair (a bimodal distribution, which has two modal peaks at each end of the continuum) or hinder such learning (a unimodal distribution, which has one modal peak at the center of the continuum). In previous studies, participants either listen to the training tokens passively (e.g., Escudero and Williams, 2014) or attentively (e.g., Ong et al., 2015) via a simultaneous auditory vigilance task during training. In this study, only the attentive listening training procedure is employed in order to be comparable to our previous studies on distributional learning of lexical tones and musical pitch (Ong et al., 2015, 2016). Learners’ performance before and after training is assessed using an ABX discrimination task^2^ in both domains. In this task, participants are required to decide whether the last sound (X) is similar to the first (A) or the second (B) on each trial. If learners engage in distributional learning, then listeners in the Bimodal condition should infer two sounds from the distribution that highlights the relevance of pitch, resulting in heightened attention to pitch after training. On the other hand, listeners in the Unimodal condition would only infer one sound from a distribution that minimizes the relevance of pitch and therefore, will not attend to pitch as much as listeners in the Bimodal condition after training. Therefore, we predict that (i) participants will show *distributional learning*, that is, those trained on a bimodal distribution will improve on discriminating items of the same domain as in training whereas those trained on a Unimodal distribution will not; and (ii) participants in the bimodal distribution groups will show *cross-domain transfer* (i.e., significant improvement on discriminating items of the *other* domain relative to training) whereas those in the Unimodal groups will not. Given that the ABX discrimination task requires listeners to use their pitch memory, pitch memory of listeners in both groups will be ascertained and controlled using a familiar song task (described below) to avoid a potential confound in the results.

## Materials and Methods

### Participants

One hundred native Australian English (AusE) listeners (85 females^3^; age range = 18–51, *M* = 21.29, *SD* = 6.04) were recruited from Western Sydney University. Forty-one were monolingual AusE listeners, and 59 were bilinguals, although none spoke a tone language. Some participants (*n* = 20) reported having minimal musical training; however, in no instance was training more than 2 years (<1 year, *n* = 1; 1–2 years, *n* = 19) and none currently practiced music. All participants reported normal hearing and no speech impairment. Participants were randomly assigned to one of four groups: Thai Unimodal, Thai Bimodal, Music Unimodal, and Music Bimodal (see Procedure of distributional learning task). These four groups are equated in terms of their age, years of musical training, proportion of female participants and monolinguals within each group (**Table 1**). Participants provided their written informed consent prior to participating and they were given course credit for their participation. The Western Sydney University Human Research Ethics Committee approved the study protocol.

**Table 1:** Demographic details of the four groups in this study.

Demographic details	Thai unimodal	Thai bimodal	Music unimodal	Music bimodal	Statistical comparison
Age	21.92 (4.582)	22.76 (8.565)	20.40 (6.576)	20.08 (2.768)	*F*(3,96) = 1.105, *p* = 0.351
Years of Musical Training	0.480 (0.7837)	0.400 (0.7071)	0.160 (0.5538)	0.240 (0.6633)	*F*(3,96) = 1.146, *p* = 0.334
Female	18	22	22	23	χ^2^(3) = 4.627, *p* = 0.201
Monolingual	9	10	10	12	χ^2^(3) = 0.785, *p* = 0.853


*The four groups are not significantly different in terms of their age and years of musical training (as determined by ANOVAs) or in the proportions of female or monolingual participants in each group (as determined by χ^*2*^ tests).*

### Stimuli

#### Distributional Learning Task

##### Thai stimuli

A subset of Thai stimuli from a previous study (Ong et al., 2015) were used as speech stimuli in the distributional learning task. The target lexical tone minimal pair was Tone 33 and Tone 241. Two native Thai speakers (one female) produced the tones in a CV syllable: the female speaker produced multiple tokens of /k^h^a33/ and /k^h^a241/ whereas the male speaker produced multiple tokens of /na33/ and /na241/. To ensure that only the pitch contour differed between each minimal pair, we first chose a base waveform from each speaker and modified the pitch contour of other tokens from the same speaker to match the base waveform. This procedure was conducted three times for each Thai word, resulting in a total of 12 Thai test stimuli (2 speakers × 2 Thai words × 3 exemplars).

The training stimuli for this task consisted of an 8-equal-step continuum of the Male /na33/–/na241/ minimal pair. The choice of this minimal pair is motivated by our previous study (Ong et al., 2015), in which it was found this minimal pair is the most difficult to perceive compared to other Thai tone minimal pairs. We reason that the participants may benefit most from being trained on the most difficult minimal pair. The intermediate tokens were synthesized by interpolating the pitch contour on Praat (Boersma and Weenink, 2013) such that along the continuum, the pitch contour transformed from Tone 33 (Token 1) to Tone 241 (Token 8).

##### Music stimuli

A subset of music stimuli from a previous study (Ong et al., 2016) was used in the distributional learning task. The target musical minimal pair was a major chord transposed to a novel scale (Dean, 2009) and a 2.5% mistuned version of that chord (e.g., Chord X–X′ and Chord Y–Y′). The frequencies of the chord were sent as MIDI via MaxMSP 5 to LogicPro 7 and realized using either a female or male choir preset (‘Astral Choir’ and ‘Choir Male Chant,’ respectively) on the Alchemy plugin. A total of four test stimuli were synthesized forming two minimal pairs: Female Chord X–X′ and Male Chord Y–Y′.

A continuum spanning the Female Chord X–X′ was also synthesized by manipulating the frequency of the middle note in eight equal steps, with Token 1 being Female Chord X and Token 8 being Female Chord X′. Following from our previous study (Ong et al., 2016), we chose this minimal pair since it was reported that this minimal pair is the most difficult to perceive among the other sung minimal pairs. It is assumed that participants may benefit most from being trained on the most difficult minimal pair.

##### Practice stimuli

To familiarize the participants with the discrimination task, a 440 Hz sinewave tone and a 440 Hz sawtooth tone, both 800 ms, were synthesized using Praat (Boersma and Weenink, 2013). In addition, the sinewave tone was used as ‘beeps’ during training as part of a concurrent auditory vigilance task (Ong et al., 2015).

#### Familiar Song Task

A familiar song task was used to measure participants’ pitch memory. Forty popular English songs as determined from a pilot study were chosen and the first 5 s of each song (i.e., the instrumental portion) was excised and duplicated. Half the duplications had their pitch raised whereas the other half had their pitch lowered, each with a transposition of either one or two semitones, resulting in four sets of stimuli (+1, +2, -1, -2) with each set having 10 songs. For the original excerpts, we manipulated the pitch upward and then downward to the same degree as their duplications in order to remove any artifacts of digital manipulation (Schellenberg and Trehub, 2003).

### Equipment

All the tasks were presented using MATLAB 2012b on an Acer TravelMate P653 laptop. The auditory stimuli were presented using a pair of Sennheiser HD650 headphones connected to an Edirol USB Audio Capture UA-25EX audio interface.

### Procedure

Participants completed three tasks (distributional learning task, familiar song task, and language and music background questionnaire), the order of which was randomized across participants. The entire experiment took approximately 50 min to complete.

#### Distributional Learning Task

There were three phases to the distributional learning task: pretest, training, and posttest. At pretest and at posttest, participants completed an ABX discrimination task (e.g., in a trial in which participants hear /na33/–/na241/–/na33/, the correct answer is A, or the first sound) for test stimuli of both domains. There were 32 trials per test phase, with eight repetitions of the four target minimal pairs (2 Thai lexical tones + 2 musical chord minimal pairs). The test trials were not blocked by test domain but presented in a randomized order for each participant. Participants were required to respond within 1 s in order to maintain vigilance and there were no replacement trials for slow responses. Prior to pretest, participants were given four practice trials with feedback.

**FIGURE 1:**
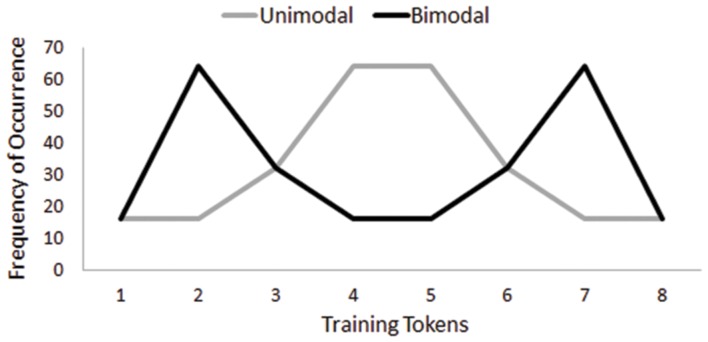
**Frequency of occurrence for each training token heard by the Unimodal condition and the Bimodal condition**.

During training, participants were randomly assigned to be trained on either Thai lexical tones or novel musical chords and then further assigned to either a Unimodal or a Bimodal condition within each domain. Listeners in both distribution conditions heard the same number of training tokens (i.e., 256) but the conditions featured different modal peaks of the distribution; listeners in the Unimodal condition heard Tokens 4 and 5 most frequently whereas those in the Bimodal condition heard Tokens 2 and 7 most frequently (**Figure 1**). Listeners in both conditions were presented with Tokens 1 and 8 (i.e., the stimuli used in pretest and posttest for the minimal pairs for both domains) the same number of times. In order to ensure that participants listened to the entire set of training tokens, they were required to complete a cover task—to mark on a response sheet with the numbers 1–288 whenever they heard a ‘beep’ by circling the sound number to which the ‘beep’ corresponded (Ong et al., 2015). A total of 32 beeps were interspersed randomly within the training tokens. The training phase took approximately 6 min to complete.

#### Familiar Song Task

In each trial of the familiar song task, participants were shown a song title and the artist who performed the song and were asked to indicate whether they were familiar with the song. If they were unfamiliar with the song, they moved on to the next trial with no replacement trials. If they were familiar with the song, they were then presented with two excerpts: an original and a transposed version, the order of which was randomized, and they were required to choose which excerpt was the original. There were two blocks of 20 trials, with one block corresponding to a pitch transposition of one semitone and the other of two semitones. The presentation order between the blocks and the trials within each block was randomized. Across all participants, the average number of songs chosen as familiar, and hence, presented to the participants, was 27.96 (*SD* = 6.61, Range = 10–40).

#### Language and Musical Background Questionnaire

In the language and musical background questionnaire, participants were asked to provide their demographic details as well as to list all the languages known to them and rate on a 5-point scale how well they (i) read, (ii) speak, (iii) write, and (iv) understand each of those languages. They were also asked to indicate whether they have had musical training and if so, the age of commencement, the duration of training in years, and the instruments played.

## Results

All participants correctly identified with 100% accuracy the ‘beeps’ in the concurrent auditory vigilance task during training, so no participant’s data were excluded from analysis. Prior to analyzing the results of the distributional learning task, we first examined participants’ performance (i) on the familiar song task, and (ii) during Pretest. Concerning the familiar song task, performance ranged from 0 to 94.44% (*M* = 64.59%, *SD* = 14.34%), with eight participants (Unimodal = 5, Bimodal = 3) meeting the Absolute Pitch criterion (i.e., accuracy of at least 85%; Deutsch et al., 2004). Importantly, the two distribution conditions did not differ overall in their pitch memory performance [*t*(98) = 1.493, *p* = 0.139]. Comparison of the two distribution conditions’ Pretest scores revealed no significant difference [*t*(98) = 0.223, *p* = 0.824]. Thus, both distribution conditions are similar in their pitch memory and in their perception of lexical tones and musical pitch; therefore, any differences between the two conditions at Posttest can be attributed to the training itself.

To deal with potential differences between Thai and music stimuli, we determined whether the stimuli from both domains are similar in difficulty by first conducting a mixed ANOVA with between-subject factors Distribution Condition (Unimodal vs. Bimodal) and Training Domain (Thai vs. Music) and within-subject factors Session (Pretest vs. Posttest) and Test items (Thai vs. Music). There was a main effect of Session [*F*(1,96) = 42.196, *p* < 0.001, $\eta_{p}^{2}$ = 0.305]: Posttest scores (*M* = 0.621, *SE* = 0.012) were higher than Pretest scores (*M* = 0.548, *SE* = 0.011). There was also an interaction between Test items and Training Domain [*F*(1,96) = 71.819, *p* < 0.001, $\eta_{p}^{2}$ = 0.428]: for those who were trained on Thai stimuli, same domain test items (Thai tones, *M* = 0.653, *SE* = 0.016) were easier to discriminate than cross-domain test items (sung chords, *M* = 0.524, *SE* = 0.018) whereas for those trained on Music stimuli, cross-domain test items (Thai tones, *M* = 0.639, *SE* = 0.019) were easier to discriminate than same domain test items (Thai tones, *M* = 0.521, *SE* = 0.016). Since Thai tones were easier to discriminate than musical chords in general, two separate sets of analyses—one for each training domain—were conducted, as participants’ (in)ability to perceive the stimuli, either during training or test, may affect distributional learning (Frost et al., 2015). For each set of analyses, we conducted a mixed ANOVA (to determine whether there was a Session ^∗^ Distribution Condition interaction) and a series of one-sample *t*-tests. The former examines whether the distribution groups significantly differed in general in their Pretest vs. Posttest scores (collapsing across the different test domains); whereas the latter provides information on whether there was *any* improvement, as indicated by whether posttest minus pretest scores (i.e., difference scores) differed from zero in each particular test domain^4^.

### Thai Tone Training Group

**FIGURE 2:**
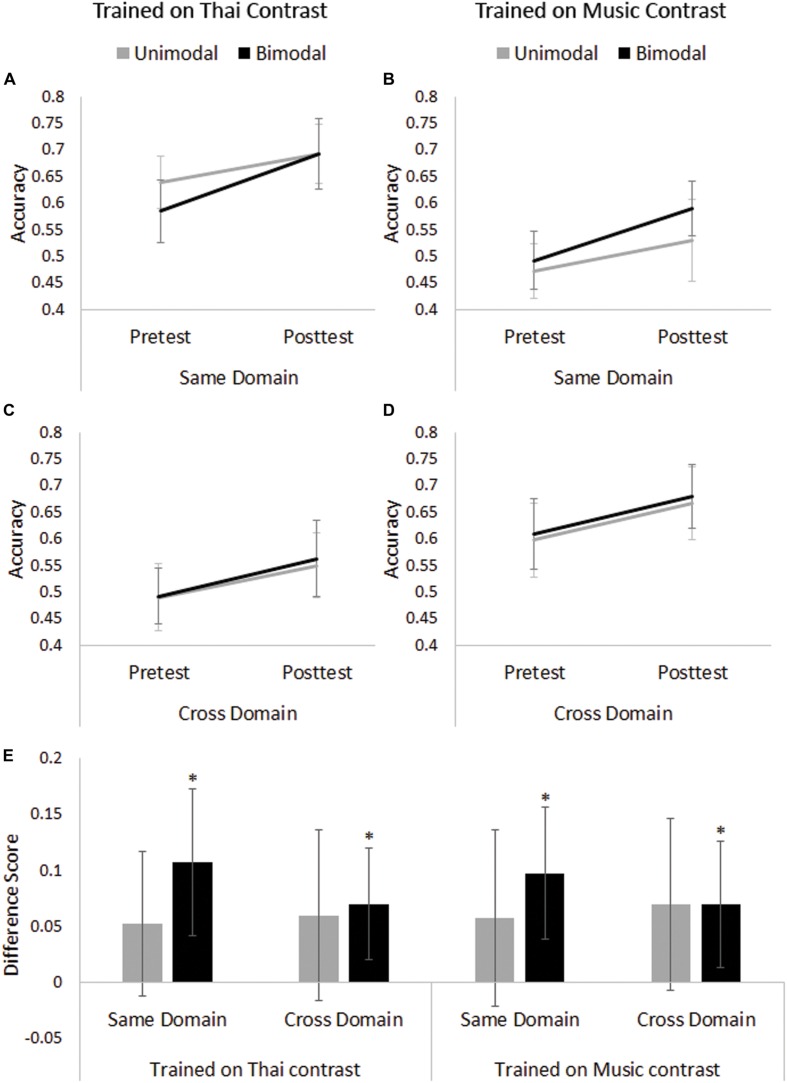
**Pretest and Posttest performance by Training Domain and Distribution Condition for **(A)** same domain test items by Thai group; **(B)** same domain test items by Music group; **(C)** cross-domain test items by Thai group; and **(D)** cross-domain test items by Music group; **(E)** difference scores (Posttest–Pretest) on the same and cross-domain test items by Training Domain and Distribution Condition.** Asterisks indicate significant improvement after Holm–Bonferroni correction. Error bars represent 95% confidence intervals.

Pretest scores in the Thai tone training group for the two distribution conditions did not differ significantly [*t*(48) = 0.857, *p* = 0.396], indicating that any difference at Posttest would be due to differences in training. **Figures 2A,C** illustrate Pretest and Posttest performance on same and cross-domain test items, respectively. The ANOVA revealed a main effect of Session [(*F*(1,48) = 21.993, *p* < 0.001, $\eta_{p}^{2}$ = 0.314]: in general Posttest scores (*M* = 0.624, *SE* = 0.016) were higher than Pretest Scores (*M* = 0.552, *SE* = 0.015). There was also a main effect of Test Domain [*F*(1,48) = 37.665, *p* < 0.001, $\eta_{p}^{2}$ = 0.440], showing that performance was higher on Thai stimuli (Same Domain; *M* = 0.653, *SE* = 0.016) than music stimuli (Cross Domain; *M* = 0.524, *SE* = 0.018). However, there was no significant Session ^∗^ Distribution Condition interaction [*F*(1,48) = 1.105, *p* = 0.298, $\eta_{p}^{2}$ = 0.023].

Following previous distributional learning studies (e.g., Escudero et al., 2011; Ong et al., 2015), we conducted four one-sample *t*-tests with Holm–Bonferroni correction on difference scores (i.e., Posttest–Pretest scores) to determine whether the improvement from Pretest to Posttest on each of the Test Domains was significantly different from zero (where zero means no improvement) for both distribution conditions. For the Bimodal Thai group, there was improvement from Pretest to Posttest on discriminating test items within the same domain [i.e., Thai stimuli, *t*(24) = 3.384, *p* = 0.002, adjusted α = 0.025, Cohen’s *d* = 0.68] *and* across to the other domain [i.e., music stimuli, *t*(24) = 2.914, *p* = 0.008, adjusted α = 0.05 Cohen’s *d* = 0.58; see **Figure 2E**]. Conversely, the Unimodal Thai group did *not* improve on test items for either domain [Thai stimuli, *t*(24) = 1.672, *p* = 0.107, adjusted α = 0.025, Cohen’s *d* = 0.33; music stimuli, *t*(24) = 1.619, *p* = 0.119, adjusted α = 0.05, Cohen’s *d* = 0.32].

### Musical Chord Training Group

Pretest scores in the musical chord training group for the two distribution conditions did not differ significantly [*t*(48) = 0.493, *p* = 0.624], indicating that any difference at Posttest would be due to differences in training. **Figures 2B,D** illustrate Pretest and Posttest performance on same and cross-domain test items, respectively. The ANOVA revealed significant main effects of Session [*F*(1,48) = 20.302, *p* < 0.001, $\eta_{p}^{2}$ = 0.297] and Test Domain [*F*(1,48) = 34.154, *p* < 0.001, $\eta_{p}^{2}$ = 0.416], mirroring the results of those trained on Thai tones. In general, Posttest scores (*M* = 0.617, *SE* = 0.018) were higher than Pretest scores (*M* = 0.543, *SE* = 0.016) and performance on Thai stimuli (Cross Domain, *M* = 0.639, *SE* = 0.019) were higher than music stimuli (Same Domain, *M* = 0.521, *SE* = 0.016)^5^. In addition, just as for Thai tones, there was no significant Session ^∗^ Distribution Condition interaction [*F*(1,48) = 0.373, *p* = 0.544, $\eta_{p}^{2}$ = 0.008].

One-sample *t*-tests indicated that, after Holm–Bonferroni correction, the Bimodal Music group improved on test stimuli of the same domain [i.e., music stimuli, *t*(24) = 3.429, *p* = 0.002, adjusted α = 0.025, Cohen’s *d* = 0.69] *and* on test stimuli of the other domain [i.e., Thai stimuli, *t*(24) = 2.562, *p* = 0.017, adjusted α = 0.05, Cohen’s *d* = 0.51] whereas the Unimodal Music group did not show improvement following training on either of the Test Domains [music stimuli, *t*(24) = 1.506, *p* = 0.145, adjusted α = 0.05, Cohen’s *d* = 0.30; Thai stimuli, *t*(24) = 1.881, *p* = 0.072, adjusted α = 0.025, Cohen’s *d* = 0.38; see **Figure 2E**].

## Discussion

The results of this study provide evidence for distributional learning of pitch-based distinctions (lexical tones and musical pitch): bimodal distribution training conditions led to improvement in discriminating minimal pairs of the same domain whereas Unimodal conditions showed suppression of any improvement due to training. This twofold outcome is consistent with our previous studies on distributional learning of lexical tones and musical pitch (Ong et al., 2015, 2016). However, we did not find significant interactions between Session and Distribution Condition in the mixed ANOVA, contrary to our previous studies (Ong et al., 2015, 2016) that reported a general (collapsing across test items) pre–post improvement for the Bimodal condition but not the Unimodal condition. This may be due to a number of reasons including individual differences in perceiving pitch, which could result in a large variance in performance (Demany and Semal, 2002; de Cheveigné, 2012). Furthermore, this study is different from previous studies in its test stimulus set variability. Similar to previous studies, we had 32 trials in each test phase. However, half the trials in this study were on two Thai tone minimal pairs (Male /na33/–/na241/ and Female /k^h^a33/–/k^h^a241/) and the other half on two musical chord minimal pairs (Female Chord X–X′ and Male Chord Y–Y′). In previous studies, all 32 trials were on four different minimal pairs of the same domain. In other words, there was less variability in the test stimuli within the same domain in this study compared to those in our previous studies, which may have weakened the effect of distributional learning. Indeed, it has been suggested that stimulus variability facilitates learning of Mandarin lexical tones, at least among high-aptitude learners (Sadakata and McQueen, 2014) and so this may account for a weaker distributional learning effect in this study relative to previous studies on distributional learning of pitch.

With regard to cross-domain transfer, the results revealed that participants who were trained on a bimodal distribution, but not those trained on a unimodal distribution, showed improvement in discriminating test items of the other domain. We propose that the observed cross-domain transfer among participants in the Bimodal conditions is due to the reliability of the pitch cue in separating the two sounds/end points in the distribution, leading to heightened attention to pitch in general. Conversely, participants in the Unimodal conditions failed to exhibit any cross-domain transfer presumably because pitch was minimized in a distribution from which only one sound was inferred and therefore those learners did not show heightened attention to pitch. The cross-domain transfer in this study was found despite qualitative pitch differences between the two domains; the lexical tone minimal pair is a difference in pitch contour whereas the musical chord minimal pair is a difference in pitch height of the middle note. Our results suggest that distributional learning of pitch-based elements (lexical tones or musical pitch) may explain the cross-domain transfer seen among ‘pitch experts’ (e.g., Pfordresher and Brown, 2009; Herrera et al., 2011; Bidelman et al., 2013; Burnham et al., 2014).

The cross-domain transfer found in this study is reminiscent of findings in speech perception research in which listeners are able to transfer a learned skill at using an acoustic cue phonemically in their native language to discriminate a non-native contrast, even if the non-native contrast is of a separate class of phonemes (Pajak and Levy, 2014). Moreover, previous distributional learning studies have demonstrated that after being exposed to a bimodal distribution spanning a consonant contrast differentiated by VOT, infant learners (Maye et al., 2008), but not adult learners (Maye and Gerken, 2001), could generalize their knowledge to a VOT contrast on a different place of articulation (e.g., improved discrimination of /g/–/k/ following bimodal training on /d/–/t/) whereas those trained on a Unimodal distribution were not able to do so. In the present study, adult learners did show generalization to a different domain, at least when a common acoustic cue is used across both domains. The difference in results between the present study and Maye and Gerken (2001) may be due to methodological differences (e.g., the use of attentive distributional learning in this study as opposed to passive distributional learning in Maye and Gerken, 2001) and/or differences in the target acoustic cue. Nonetheless, our results suggest that distributional learning is generalizable to a different domain.

While we have found some evidence of transfer between speech and music, these results raise the question of the extent to which the signal needs to be similar in order for transfer to occur. One possibility is that the learner cognitively compares the acoustic signals and if the relevant features are deemed similar enough, then the same processing will be applied to both signals. This would be in line with the proposal that speech and music, despite having very different representations and output, share similar processing mechanisms since both domains share similar acoustic cues (Patel, 2008). The proposal is complemented by the fact that the auditory system appears to be agnostic with respect to the nature of the signal in its early stage of processing (i.e., in the brainstem; Krishnan et al., 2010; Bidelman et al., 2011) and presumably not until much later during processing is the signal’s cognitive function defined. Therefore, it could be argued that cross-domain transfer may be dependent on whether the features of interest that define the signals are deemed similar enough by the learner.

On the other hand, it is possible that participants in this study did not treat the stimuli (lexical tones and sung musical chords) as belonging to different domains, and therefore did not actually show any cross-domain transfer at all. While, acoustically, there appear to be some differences between speech and song—for example, articulation is often more precise in speech than in song and vowel duration tends to be longer in song than in speech (Seidner and Wendler, 1978, as cited in Merrill et al., 2012)—these characteristics may not be as evident if the signal is short in duration, such as in the case of isolated lexical tones or a sung note or chord. Moreover, participants’ functional interpretation of the signal may change under certain conditions—for example, a speech signal may suddenly be interpreted as someone singing when presented repeatedly (Deutsch et al., 2011). Thus, lexical tones, when presented repeatedly and in an isolated context, could be interpreted as someone singing and perceived as music-like. Indeed, tone language listeners but not non-tone language listeners show left lateralization of processing lexical tones whereas both groups show right lateralization of processing hummed version of lexical tones (Van Lancker and Fromkin, 1973, 1978; Van Lancker, 1980). This suggests that tone-language listeners treat isolated lexical tones to be speech-like more than do non-tone language listeners, who may treat isolated lexical tones to be more music-like. In contrast, Burnham et al. (2014) found that non-tone language non-musicians seem to treat lexical tones to be speech-like, at least when lexical tones are presented with two other sets of stimuli: low-pass filtered lexical tones (i.e., tones that do not convey any phonological elements of speech and consist only of frequencies below 270 Hz) and violin notes that approximate the pitch contour of the lexical tones. In other words, it is possible that the participants in Burnham et al. (2014) may have treated lexical tones to be speech-like *relative* to the other stimuli that are presumably interpreted as more music-like. Thus, it remains plausible that the participants in the present experiment may consider isolated lexical tones and synthesized sung chords to be from the same domain.

To eliminate the possibility that participants in the present study perceived lexical tones and sung chords to be from the same domain, we conducted a stimulus identification pilot study with a different set of participants from the same population (i.e., AusE non-musicians, *n* = 23). Participants were required to decide whether the signal was ‘spoken’ or ‘sung’ and then rate their confidence on a 5-point scale (1 = “somewhat confident” and 5 = “very confident”) without any feedback. On every trial, we scored the participants based on how confident they were: participants were given a positive score that corresponds to their confidence rating if they were correct and a negative score that corresponds to their confidence rating if they were incorrect. Two overall scores were obtained for each participant, one corresponding to each domain. The results revealed that participants perceived isolated Thai lexical tones as ‘speech’ and synthesized sung chords as ‘sung’ at rates above chance [Thai lexical tones: *t*(22) = 5.052, *p* < 0.001; sung chords: *t*(22) = 20.607, *p* < 0.001]. However, performance on lexical tones (*M* = 1.93, *SE* = 0.38) was significantly lower than on sung chords [*M* = 4.07, *SE* = 0.20; *t*(22) = 5.745, *p* < 0.001], suggesting that as we suspected, isolated lexical tones are more ambiguous for non-tone language non-musicians than are sung chords. Taken together, this suggests that despite being less confident and less accurate in identifying lexical tones as spoken, the participants treated the stimuli as intended, which provides us with some assurance that the participants in the main experiment treated the isolated lexical tones as speech and therefore indeed demonstrated cross-domain transfer^6^.

## Conclusion

Our results indicate that naïve learners show distributional learning of lexical tones and musical pitch, thus replicating our previous results (Ong et al., 2015, 2016). More importantly, we demonstrated that distributional learning may play a role in cross-domain transfer: in this study, cross-domain transfer was only observed among learners who inferred a pitch distinction in the input (such as learners in the bimodal condition), which presumably led those learners to show heightened attention to pitch in general. These results are indicative of cross-domain transfer, especially given that a follow-up study provides evidence that the lexical tone and musical chord stimuli were identified accurately according to their specific domain. The effect of distributional learning appears to be smaller in this study than that in our previous studies. This may be due to large individual differences in pitch perception and the lack of variability in the test stimuli within each domain. Nonetheless, our findings suggest that acquiring speech or musical items based on subtle pitch differences may lead to cross-domain transfer of pitch.

## Author Contributions

JO posed the research question and designed the experiment with useful feedback from DB, PE, and CS. JO prepared the stimuli, programmed the experiment, recruited the participants, conducted the experiment, and performed the data analyses. JO prepared the manuscript with valuable input from DB, CS, and PE.

## Conflict of Interest Statement

The authors declare that the research was conducted in the absence of any commercial or financial relationships that could be construed as a potential conflict of interest.

## Footnotes

1 In this paper, lexical tones are described using Chao values (Chao, 1930), where the numbers 1–5 indicate the relative height and contour of initial and final and sometimes the medial values of the tone with 1 being the lowest and 5 being the highest. For example, /ma55/ is a high level tone whereas /ma51/ is a high falling tone.

2 One may be wary of a discrimination task as a means to investigate learning of categories, instead of using both discrimination and identification tasks. In this study, where speech and music sounds are used, identification tasks using (arbitrary) labels is not ideal as precision in sound-to-label mapping may be different for speech and music, particularly since labels are not typically given to musical pitch (with the exception of absolute pitch possessors). Note that category formation does not necessarily imply ‘categorical perception’ of the newly formed categories, that is, a decrease in within-category and an increase in between-category sensitivity as a result of warping due to an abstract category. For instance, vowels are considered as separate categories even though vowels are typically perceived in a continuous manner (e.g., Fry et al., 1962).

3 The larger number of female participants is representative of a larger female cohort among Psychology undergraduate students in Western Sydney University. A previous study that examined gender differences in perception of naturally produced vowels (which implies the presence of multiple acoustic cues) using a distributional learning paradigm did not reveal any gender differences in initial perception, although dividing the participants into high vs. low initial performance did reveal gender differences in integrating new cues (Wanrooij et al., 2013). Unlike the previous study, only one cue is present in each minimal pair in the present study, and therefore gender differences in integrating multiple cues is not an issue. Furthermore, there is no *a priori* reason to expect gender differences in pitch perception, though future research could investigate this to be sure.

4 It should also be noted that given the current experimental design (pretest–training–posttest), some improvement is to be expected due to practice, irrespective of the distribution encountered during training (Ong et al., 2015). However, if the Unimodal group fails to show significant improvement *despite* the pretest–training–posttest design, then this lack of improvement due to practice can be taken as a stronger evidence of distributional learning over and above the improvement that is predicted for the Bimodal condition.

5 The somewhat counterintuitive finding that performance on the cross-domain test items is higher than the same domain test items for the music training group reflects the fact that our Thai stimuli were easier to perceive than the music stimuli, a finding that was found consistently in all our analyses in this paper. One possibility for such finding may relate to the unfamiliar nature of Thai lexical tones relative to sung chords to non-tone language listeners, who may not be subjected to interference for the Thai tones due to their unfamiliarity.

6 It is possible that our choice of sung chords as a musical medium may be considered speech-like and hence bias the results toward cross-domain transfer. While that is certainly a possibility, the sung chords used in this study consist only of vowels which do not have any lexical meaning. Furthermore, as revealed in the stimuli identification task, the sung chords are indeed perceived to be sung and distinct from speech. Therefore, the sung chords are perceived to be musical by our participants and presumably have the same status as musical tones produced from musical instruments. The experiment could certainly be repeated with tones from musical instruments and it is certainly worth investigating in the future whether there might be differences in cross-domain transfer if the musical stimuli are sung or musical instruments.
